# The ethics of knowledge sharing: a feminist examination of intellectual property rights and open-source materials in gender transformative methodologies

**DOI:** 10.3389/frma.2024.1321302

**Published:** 2024-10-03

**Authors:** Leah Goldmann, Alice Welbourn, Diane Gillespie, Nastnet Ghebrebhran, Lufuno Barro, Sara Siebert, Hawa Kagoya, Lori Michau, Anjalee Kohli, Tina Musuya, Sonia Rebecca Kusiima

**Affiliations:** ^1^Raising Voices, Violence Against Women Prevention, Kampala, Uganda; ^2^Salamander Trust, London, United Kingdom; ^3^University of Washington Bothell, Interdisciplinary Arts and Sciences, Psychology, Bothell, WA, United States; ^4^IMAGE (Intervention with Microfinance for AIDS and Gender Equality), Johannesburg, South Africa; ^5^Independent Consultant, Kampala, Uganda; ^6^Independent Consultant, Washington, DC, United States; ^7^Social Development Direct, Gender-Based Violence, London, United Kingdom; ^8^TUJAYEPA, Kampala, Uganda

**Keywords:** gender transformative approach, open source (OS), intellectual property (IP), feminist methodologies, social norms change

## Abstract

Debates on intellectual property rights and open source frequently stem from the business sector and higher education, where goals are typically oriented toward profit, academic status, credit, and/or reputation. What happens if we reconsider the ethics of intellectual property rights and open source when our driving motivation is advancing women's health and rights? How does this prioritization complicate our assumptions of copyright and open access? How can we embark on a journey that validates the complex realities of multiple stakeholders who have good intent, but do not always consider the unintended impacts and the broader power dynamics at play? This paper explores the tensions and nuances of sharing methodologies that aim to transform harmful gender norms in an ecosystem that does not always consider the complex challenges behind intellectual property and open-source material. As a thought-collective dedicated to using a feminist approach to unpack and promote the principles of ethical, effective, and sustainable scale, we hope to underscore how the current research and debates on intellectual property rights and open-source material have good aims but may also fall short in encompassing the realities of gendered social norms change in and with communities around the world. We conclude with key recommendations for donors, researchers, International Development Corporations, International Non-Governmental Organizations, and those interested in using or adapting dynamic, gender transformative materials created by others.

## Introduction

The Community for Understanding Scaling Processes (CUSP) is a thought-collective of organizations, including the Center for Domestic Violence Prevention (CEDOVIP), the Center on Gender Equity and Health—UC San Diego, Intervention with Microfinance for AIDS and Gender Equity (IMAGE), Oxfam, Raising Voices, Stepping Stones, Tostan, and Tujayepa (formerly CFAR-Uganda), focused mainly across Sub-Saharan Africa that uses practice-based learning to inspire conversations and influence action on ethical and effective scale of social norms change programming. CUSP is composed of organizations and individuals who have created, documented, implemented, adapted and/or directly participated in community-based gender-transformative social norms change methodologies that are often recommended for scale because of their demonstrated impact through evaluations and randomized controlled trials (RCTs). Each member of CUSP has significant experience in understanding the challenges and opportunities of scale as our work is used in new contexts. Since 2016, CUSP has led dialogues and produced materials that share our experiences with scaling these respective approaches, turning a critical eye toward reducing harm and prioritizing benefits to women and communities. Over the past 2 years, CUSP has explored the concept of feminist scale, inspiring an examination of how open-source materials and the sharing of intellectual property can align with feminist principles.

The aim of this paper is to contextualize the issues that social norms change organizations may face when sharing materials openly. It is primarily written for consideration by donors, researchers, International Development Corporations (IDCs), International Non-Governmental Organizations (INGOS) and others interested in using or adapting dynamic materials created by others. We examine the intended benefits of intellectual property rights (IPR) and open source for social norms change methodologies and then explain the difficulties that have arisen that have caught us off guard and unprepared for the repercussions. Through our analysis, we have come to see that those difficulties are often related to the policies and practices of IPR, which can be misaligned with promoting public health. In tracing the history of IPR, we show how legal protections require codification of “products” or “ideas” to secure authorial integrity, but they also get linked to profits and capitalist mindsets that prevail in the Global North. So, when advised to look to IPR for help, we find that they don't always function for us. When looking at the literature on open source, we find ourselves in a similar conundrum, where advancement in technology around making materials open source can—but does not always—support the responsible use of these methodologies.^1^

We note how the educational materials produced by our programs can be used as products for distribution—finished written manuals, with an evidence base for their effectiveness in reducing violence against women (VAW). Yet for some of us, our published materials are only the *beginning* of the journey which makes them useful in effecting gendered social norms change in and with communities. Just like a school textbook, which is only as good as the quality of its use in the classroom with and by students, our own materials need considerable support to be used effectively. Our materials are designed to promote *interactive* communication *between* individuals and groups, through the active, collective creation of verbal and visual drama, public and private discussions, dance, art, poetry, protest, storytelling, song, and other forms of activism. While reading a book or manual is a one-directional process, communication, sharing, and learning are iterative and multi-directional; this approach sparks community agency through dialogue, reflection, role-plays, debate, consensus, decisions, and actions that lead to change. Community members, facilitators, activists, and the organizations that received funds from the donor for this work, and the funders themselves, all have vital roles in this chain of events from written word to community action. The qualities of *who* they are and *how* they perform have a critical effect on the quality of changes that can take place in the community. The inanimate written text of the materials comes alive through a process that requires sensitivity to and knowledge of context, training, mentoring and support.

Therefore, in this paper we argue that the policies and practices governing IPR enlisted by those who promote and distribute gendered norms change materials for mass scale-up presents us with a limited frame of reference—a frame which conflicts with our feminist values. We have previously described the feminist principles which enable feminist movement-building at scale: balancing power to advance the collective good; seeking to upend inequities and foster change at its root; building out intersectional approaches that support women in all of their diversities, questioning hierarchies within communities and across organizations in the aid sector; valuing all voices; strengthening self and collective care practices; and cultivating a relational approach to our work through collaboration, accountability, and joy (Community for Understanding Scale Up, 2022). These principles are realized through essential elements that cultivate the foundation for feminist scale: community-led initiatives that value local knowledge, agency and vision; human rights-based approaches that address structural inequities including colonialism and racism; values-driven organizations which embed feminist values into all levels of their work; grounding efforts in local context that addresses political, social economic and structural realities; iterative learning that allows for flexibility and adaptation, and which values practice and research-based learning and; responsive funders that support organizations through long-term, core support (Community for Understanding Scale Up, 2022).

However, we have seen that stakeholders sometimes overlook the deep investment and experience required to produce methodologies that address root causes, ensure inclusivity, value local knowledge and agency, and question hierarchies. The communication needed to bring materials to life safely and effectively in communities requires ongoing collaboration, mutual learning and sharing beyond the written text; yet this can be replaced by a process of mass distribution which diminishes the human connections involved and undermines these foundational elements. We hope to encourage donors, researchers, IDCs, INGOs, and others to adopt a more nuanced outlook on IPR and open-source issues in the context of scaling gender transformative social norms change programs and methodologies, particularly one that reflects feminist principles, prioritizes the safety of communities in which these methodologies are implemented, and inspires generative innovation.

## Intellectual property, global development, and CUSP's application

The full history of IP is beyond the scope of this paper, and, in a general sense, all agree that creators need protection from theft and/or misuse. But two historical developments are relevant for the challenges that some CUSP programs currently face: first, modern IP laws and regulations derive from Western capitalistic societies to protect private property and profits associated with that property; second, the business model for IP came to dominate the medical world in the North, especially the USA. The early models of international aid tended to be medical and thus patents and licensing came into international development with a set of understandings about what could be protected and what not. Programs such as CUSP's, far from “market-driven” delivery models of aid, found that this conceptualization of IP can lead to unintended consequences for communities, our organizations, and the broader field aiming to transform gendered norms. For purposes of this paper, we refer to IP when describing the content, material, and knowledge that is under protection, while IPR refers to the rights, policies, and laws that govern the protection of intellectual property.

### Defining IPR and its relationship to human rights

IPR concerns have been documented since at least 1474, and have increased dramatically with industrialization and, more recently with neoliberalism and capitalism (Drahos, 1999). Modern understandings of IP are drawn from Western notions of ownership, innovation, and “romantic individualism.” Regimes of IPR have closely aligned with access to education, money, and/or land and subsequently strengthened through colonial power (Carpenter, 2004). The World Intellectual Property Organization (WIPO) divides intellectual property into two categories: industrial property and copyright. The former includes patents, trademarks, and industrial designs, while the latter includes literature, artwork, and software (WIPO, n.d.). With this limited scope of definitions, other forms of knowledge have been excluded from intellectual property (IP), such as the knowledge derived from plants and animals in medical treatment and foodstuffs among Indigenous groups (Carpenter, 2004; Gana, 1996; OseiTutu, 2016; Yu, 2016). Thus, IPR's protection of certain types of knowledge has simultaneously delegitimized other types—particularly knowledges claimed by those who have fewer resources and limited access to powerful institutions (Carpenter, 2004; Gana, 1996).

Various human rights documents protect property rights and knowledge production. Article 27(2) of the Universal Declaration of Human Rights (UDHR) states “Everyone has the right to the protection of the moral and material interests resulting from any scientific, literary or artistic production of which he is the author”. The International Covenant on Economic, Social and Cultural Rights (ICESCR) mirrors this language almost identically in Article 15(1). However, “property” and “intellectual property” are not defined under these documents, nor is there clear guidance on how this protection is to be achieved, often delegating national legal infrastructure to establish the scope of property rights (Cornides, 2004; Geiger, 2015).

On the one hand, a strong IPR system can promote economic and social development. Kabanda (2016) argues that while the African continent's extensive variety of culture and arts has had a major influence on the quality of life for Africans, a weak IPR regime prevents countries from fully profiting from their creative potential. Kabanda (2016) analyzed the crafts industry as a central contribution of GDP for countries such as Burkina Faso, Morocco, and Ghana, which serve as major craft hubs that have achieved global recognition of their creativity. As a result of globalization, there have been rising concerns about industrialized counterfeiting, with artisans unable to compete with the cheaper, mass production of crafts that imitate their designs. However, a 1960s Ghanian law registering textile designs excluded the Kente because of their collective, cultural significance in the country, leading to leading to what has been described as “economic colonization” (Kabanda, 2016). Ghanaian artist Bobbo Ahiable's Kente reached international appreciation in institutions such as the Smithsonian, yet a lack of IP protection enabled a major U.S. corporation to reproduce and profit off of his design. Counterfeiting and economic colonization more broadly could be mitigated through stronger copyright laws that prevent corporations from profiting off of artisans' work.

Yet, this case study raises broader concerns around the incompatibility between an IPR regime that prioritizes the individual over the collective—a value deeply integral to many non-Western countries— and how wealthier countries can take advantage of this gap. IPR critics argue that Western-style laws have not been effective in the Global South, leading to rising medicine costs and obstacles to accessing local knowledge that could better address health disparities (Sekalala et al., 2021). Others such as Ruth Gana, have gone further and contend that the concept of IPR is incongruous with collective development and human rights. For example, copyright “denies the reality that progress in every society relies partly on the heritage of others and the very act of creating is stimulated by what has preceded” (Gana, 1996). Arguably, IP thrives primarily in countries that promote individualism and accumulation of private wealth. Betty Mold-Iddrisu, a Ghanian lawyer and politician, asserts that the IP protection regime fails African women, preserves elitism, and is incompatible with collective systems of creativity in Africa. For instance, in the textile design industry, indigenizing efforts to expand the economy in post-colonial West Africa excluded women's contributions (Mould-Iddrisu, 1997; Kwame Nkrumah Festival, 2021). In fact, several UN Special Rapporteurs and General Comments on the “rights to food, education, health, science and a cultural life” have recognized an “incongruous partnership between intellectual property and human rights,” noting that copyright laws have enabled corporations to, among other rights violations, hold a monopoly over textbook production, deny affordable health care, and conflict with the right to food under IPR provisions for plant and food varieties (Dutfield and Suthersanen, 2020).

Decolonial global health scholars envision a world where healthcare, specifically access to medicine, is free from intellectual property law. Sekalala et al calls for “reparative redistributing of resources in global solidarity, shifting vaccine access from a charitable plea to a legal obligation, increasing manufacturing capacity in the Global South and clarifying human rights responsibilities of pharmaceutical corporations themselves” (2021). Such a framework would differ from current international IP rules by not placing restrictions, such as specific regulations or loan acquisitions, to access services (Sekalala et al., 2021). For instance, in the wake of COVID-19, the ICESCR confirmed that states have a duty to prevent IP legal regimes from interfering with the right to the “enjoyment of economic, social and cultural rights,” including the protection of public health (Committee on Economic, Social, and Cultural Rights, 2021).

A common argument in favor of IPR is that copyright and patents drive innovation. However, evidence for this argument is inconclusive; there has been limited empirical evidence to explore the relationship between innovation and IPR, particularly when comparing industrialized countries to countries in the Global South (OseiTutu, 2016). In fact, some scholars have used the term “faith-based IP” to describe the unbridled faith in strong IP protection among proponents, where IP is justified as a “moral end in itself” (Lemley, 2015). While there is an impressive literature on IP, “none of these studies resolves whether patents have a net positive effect on innovation, much less their net welfare effect, or whether alternative innovation incentives such as grants, prizes, and tax credits are inferior” (Ouellette, 2015). In some cases, IPR has had a palpably negative effect worldwide, such as through the biotech corporation Monsanto, which operated under US-patent laws around the world, ignoring national legal infrastructures, devastating- local farmworkers' livelihoods, and harming biodiversity (Peschard and Randeria, 2020). Overall, the relationship between IPR and innovation varies depending on countries in question; the oft-cited argument of innovation expressed in favor of IPR arguably advances the political agenda of leaders and corporations in those countries who benefit the most (Geiger, 2015).

Thus, as demonstrated by this selected collection of literature, there is a complicated interaction between human rights and IPR in theory and in practice. Spina Alì's (2020) taxonomy draws upon considerable literature regarding IPR and human rights (e.g., Dutfield and Suthersanen, 2020; Geiger, 2015; Torremans, 2019) by summarizing three forms of legal interactions: recognition, conflict, and cooperation. Under “recognition,” the law acknowledges the human rights dimensions of IP. For example, the United Nations (UN) recognizes “the right of everyone to benefit from the protection of the moral and material interests resulting from any scientific, literary or artistic production of which he [or she or they] is the author”. Next, “conflict” commonly illustrates the possibility of competing interests between IP and human rights. Court decisions typically resolve these cases by attempting to balance the incentives of the IPR system with the public's freedom of expression, freedom to education, right to health, and the right to enjoy the arts and the benefits of scientific innovation (Spina Alì, 2020). Lastly, “cooperation” refers to human rights and IPR acting in synergy, such as through complementarity, whereby both IPR and human rights are separated but interconnected aspects of the same issue (Spina Alì, 2020). Through this framework, we can acknowledge that the relationship between IPR and human rights ranges across a spectrum, from clashing to working together with mutual respect.

### Intellectual property in global development

In global development, conversations on IPR are primarily restricted to the health sector. Multilateral institutions such as the UN and World Trade Organization (WTO) have largely supported universal IPR under the theory that such access could reduce barriers to medicine access and assist LMICs. The World Intellectual Property Organization (WIPO) is assigned with protecting IP within countries and international organizations (WIPO, n.d.) with the view that IP is a “force for innovation and creativity” (Leal et al., 2014). Critics argue that the agency did not fundamentally take development implications into consideration for 40 years. Countries in the Global South were responsible for adapting to strict copyright and patent standards set by the Global North, denying them the process of developing their own regulations (Dutfield and Suthersanen, 2005). Others have accused WIPO of promoting a “one-size fits all” approach to IP rules, furthering inequities between the Global South and Global North (Dumont and Fastame, 2009; Leal et al., 2014; Yu, 2016).

In addition to WIPO, the WTO's mandate is to oversee global trade between countries, including IPR through the WTO Agreement on Trade-Related Aspects of Intellectual Property Rights (TRIPS), which came into force in 1995, setting minimum standards for national protection of IPR and included WTO- enforcement mechanisms such as trade sanctions (Guaran, 2009; Kingston, 2011). Kingston argues that TRIPS was devised by powerful countries to have further control over global innovation, with many countries in the Global South resisting the TRIPS agreement out of fear that it would increase prices and negatively impact their countries' industries (2011). Through a review of IPR on access to medicines following 25 years of TRIPS implementation, researchers analyzed patent data from the African Regional Intellectual Property Organization. Findings revealed that the IP frameworks enforced under these organizations “are inconsistent and misaligned with the TRIPS Agreement and are more onerous than the minimum standard provided by TRIPS,” further stating that there has not been a differentiation of obligations for countries designed to benefit from TRIPS and countries in the Global North (Motari et al., 2021). These findings echo critiques of WIPO, which in practice, still maintains a “one-size fits all” approach.

Some scholars argue that TRIPS views health considerations as exceptions to IPR, requiring nations to defend their prioritization of human development “as justified, despite the obligation to protect intellectual rights” (OseiTutu, 2016). For instance, cigarette companies in Australia reasoned that a national marketing campaign to limit cigarette use, called “Plain Packaging Legislation,” violated their trademark rights because it limited the use of logos and imagery on tobacco. The cigarette companies claimed that the legislation was a violation of Australia's intellectual property obligations under WTO agreements (OseiTutu, 2016). In 2018, a WTO Panel dismissed all claims by the cigarette companies, declaring that public health protection was a legitimate reason to impose special requirements under TRIPS. Tobacco control outweighed the effect of trademark use, demonstrating WTO's impact on national public health initiatives (Mitchell, 2022). Given the interaction between IPR and public health in instances such as these, the implications for the global health sector are evident.

The HIV and AIDS crisis launched conversations at the international level around vaccine access and compelled urgent reframing of policies related to medical patents. The Doha Declaration in 2001 permitted compulsory licensing, enabling generic manufacturers to produce more affordable patented medicine (Kingston, 2011). Prior to TRIPS, Brazil was the first country in the Global South to develop a comprehensive program for accessing affordable HIV antiretroviral treatment, but growing pressure from wealthy countries to strengthen its national patent laws forced the country to redirect a majority of their program budget to cover the fees associated with the patent laws (Hoen et al., 2011). The Doha Declaration confirmed TRIPS' commitment to prioritizing health as a human right over IP, at least on paper, particularly in light of the HIV crisis and lack of access to affordable treatment (Barbosa et al., 2007). Critics claim that, despite this welcome addition in theory, Doha did not consider the impact for countries without national manufacturing capacity, thus limiting their ability to procure an affordable version of the drug (Haakonsson and Richey, 2007; Kingston, 2011). These examples point to the complex interactions between IPR and human rights and the failure of WTO's mandate to meet diverse needs of all countries under WTO's mandate—unsurprisingly, they do not speak to the challenges described in CUSP's context.

More recently, the People's Vaccine Alliance (PVA), a coalition of organizations and activists, advocates for a people's COVID-19 vaccine through policy proposals such as “patent pools, compulsory licensing, government use or crown use, public sector licensing, patent pledges, open licensing, and open innovation” (Rimmer, 2022). Although PVA advocates for vaccination as a public and global service, it nonetheless campaigns for *safe* distribution of vaccines by qualified healthcare professionals to comply with the “Do No Harm” principle, by maintaining quality control of vaccine roll-out programmes. So, proponents are advocating for increased *quantity* of access, whilst also ensuring that *quality* of delivery is maintained. This example speaks more directly to CUSP's experience, given our experiences as creators of gender-transformative change curricula that are being scaled up, where we sometimes see quantity (“numbers served”) prioritized at the expense of quality programming.

In summary, the tracing of policy on global trade and patented medicines reveals a problematic history, one which has often prioritized wealthy nations and individuals in the Global North at the expense of the right to health for those living in the Global South. All of these examples stand apart from protection of CUSP's work; and yet, even given the troubling outcomes within each one, the policies and legal histories are used as the background against which protection of our materials must be considered. In relation to broader conversations on decolonization in the global development sector and the power grip that wealthier nations hold, it is essential to critically analyze how the rules and regulations governing global health have excluded, overlooked, and/or ignored the perspectives and realities of what is happening at national and local levels. This evolution of policy provides the basis for how we understand IPR in global development, creating conflict with several of the key values of feminist scale we have described, such as balancing power between individuals and across the South-North divide, addressing the political nature of social norms change work (as opposed to a purely technical one), and infringing upon the human right to health.

### Intellectual property rights and CUSP's experiences

Like the taxonomy set forth by Spina Alì (2020), CUSP members have found that there are diverse ways of framing the interaction between the ethics of IPR and development, from cooperation to conflict. In an ideal world, we could honor the relationship between IP and human rights, where well-adapted CUSP methodologies are used with fidelity to their principles and structure with maximum benefit to communities, while also respecting creative integrity. Yet, we often find ourselves in relationships that undermine quality programming. Most CUSP member organizations have openly shared their materials, and some have even built infrastructure to assist with new adaptations and scaling of their methodologies through feminist partnerships in the spirit of advancing mutual shared goals and collaborative learning.

The Institute for Reproductive Health (IRH), for example, had organizational structures and funding mechanisms that meant their materials were designed from the outset to be used by many organizations, particularly as recipients of bilateral funding. IRH is a research institute affiliated with Georgetown University that aims to advance family planning globally. IRH, Pathfinder and Save the Children developed The Gender Roles, Equality, and Transformation (GREAT) project. Designed with scale in mind, GREAT aims to promote gender equitable attitudes and behaviors among adolescents (ages 10 to 19) and their communities. Many practitioners do not have the time, funding, or capacity to develop or innovate, while others seek to avoid replicating what already exists in developing programs in every setting in which they work, and still others want to use evidence-based materials, so they adopt existing, evidence-based programs such as GREAT. Based on these processes, the GREAT program was designed with the intent of being used by many organizations in diverse contexts, in tandem with ethical adaptation.

However, other CUSP members have had more difficult engagements with IPR, such as Salamander Trust and their *Stepping Stones* program. *Stepping Stones*, originally developed in Uganda, uses a structured curriculum to support participants across 4 peer groups—based on gender and generation—to analyze the complex sexual and reproductive health and rights-related issues they face in the context of VAWG and HIV. The peer groups work separately and together to build community-wide consensus to overcome them to achieve gender-transformative social norms change. The curriculum has been designed for constant adaptation and updating, to align with and address ongoing scientific advances and emerging topical issues. Yet, organizations have misused Stepping Stones' IP in a way that results in reduced-quality outcomes for communities. For example, Gardsbane and Bukuluki (2023) found that a USAID-funded implementation of *Stepping Stones* counseled adolescent girls and young women on how to alter their behavior in order to minimize tension with their male partners, rather than shifting harmful gender norms. This work was framed as an adaptation of Stepping Stones' IP, and yet violated some of the fundamental elements of the program.

Similarly, Raising Voices was met by a team of international lawyers and legal documents that offered no protection for Raising Voices' IP, when presenting a multilateral agency with a simple Memorandum of Understanding (MoU) outlining terms of ethical adaptation and use for *SASA!. SASA!* is a holistic community mobilization approach to preventing VAW that involves addressing deep-rooted norms on gender and power, requiring about three years of meaningful community engagement to yield impact. Because the materials were in the public domain, the multilateral agency moved ahead to use Raising Voices' material while refusing basic safety standards, putting Raising Voices in a position where they felt they had little choice but to engage under the multilateral organizations' protocol. Since the *SASA!* RCT results were released, there has been a sharp rise in requests for ‘crisis' technical assistance attempting to (re)train implementing staff after they have begun programming or to redesign programs due to negative feedback, poor results and even increases in VAW. In many cases, there is no funding for this assistance. Because of the concern that the misuse of materials would harm women and communities in contexts such as these, CUSP members have abandoned their efforts to protect their IP to support safer program design and implementation.

CUSP's adverse experiences with IP motivated our collaboration and activism. In some instances, there is clear plagiarism, whereby organizations and/or individuals publish materials on their websites with no reference to the originators who created them, even renaming the program in a handful cases. Consequently, originators of the methodology expend staff time and energy dealing with the burdens and liabilities that accompany the misuse and misrepresentation of their materials, pulling them from planned programming because funders and implementers may not have adhered to guidance on adaptation, fidelity, and/or methodology use. This is a lose-lose situation at all levels: potential partners miss out on key insights and critical program updates from originators; originators and other quality adapters miss out on invaluable opportunities for ongoing mutual learning, collaboration, and innovation; the entire sector loses because movement building flourishes within an ecosystem of open learning and accountability; and, most importantly, communities can be negatively impacted by poorly adapted and implemented programs.

Chon argues that IP should be modified to “global knowledge governance” that includes, among other knowledge, tacit knowledge, and social norms (Chon, 2019). CUSP has written extensively about varied challenges we have experienced with others' use of our respective materials to enable communities to deliberate about harmful and beneficial norms so that they can decide whether they want to change them (Goldmann et al., 2019). Chon envisions a “social justice-driven” IP, rather than a “market driven one.” Relevant to CUSP's experience, Chon uses case studies of “regime-straddling mechanisms” which cut across “development policy and public and private sectors,” such as innovative medicines initiatives, which use “patent pooling” in public health to deliver affordable medicines around the world (as we described above with vaccines). Members of CUSP have attempted a similar solution for methodology use through the pooled training and implementation of some of their methodologies, enabling broader access and collaboration.

In Timor-Leste, the Asia Foundation (TAF), in partnership with the Prevention Collaborative, engaged in a process to translate, adapt, and evaluate a contextualized violence prevention curriculum called NeNaMu that combines the basic structure of *Stepping Stones* with additional content from *SASA! Together* and other materials. TAF, already a long-time partner to Raising Voices, entered an MoU with Salamander Trust and Communicating for Action and Results Uganda (CFAR) to facilitate initial intensive training and provide ongoing advisory support throughout the project to ensure that the material has been adapted ethically and appropriately for the new context. In this partnership, Salamander Trust and CFAR-Uganda were contacted in the beginning of the project, have been consulted throughout, and their time has been compensated (Prevention Collaborative and Nabilan, 2021). One result of this fruitful collaboration is that TAF entered a partnership with Estrela +, the network of people living with HIV in Timor Leste, to increase understanding of the linkages between VAW and HIV in Timor Leste, and to advance the principles of feminist scaling through movements, as we described in 2021 (see Community for Understanding Scale Up, 2021).

In sum, CUSP does not necessarily advocate for or against IPR, but we share our experience on IPR to increase awareness of the issue and call for a broader social justice-driven IP rather than a market-driven one. To promote the spirit of evolving knowledge and the conviction that innovation and knowledge are the production of many, CUSP has looked beyond the use of IP and legal enforcement to protect our materials, while also enabling their productive evolution and fostering innovation. Such alternatives under consideration include creating a practitioner-based knowledge exchange to build mutual accountability to and respect for feminist methodologies and principles; providing a protection clause to safeguard against misuse of material; as well as developing clear guidance for feminist scale up based on our past publications. IP is rarely discussed in the production of social norms change methodologies yet poses immense challenges—and opportunities—for the future of scaling social norms change. While we may have unique approaches to IPR, our collective voice honors acknowledgment, upholds safety and generates innovation.

## Open-source content, global development, and CUSP's application

As organizations, we have had different experiences in the level of “openness” of our approaches, but the principle of “open source”—to make work accessible and more egalitarian— align with our feminist ethics; it honors our belief in the feminist principle that our work is in service of others with our efforts being for the good of all, not the advancement of an individual or organization. Unfortunately, we have also seen our methodologies used in ways that caused harm to women and communities, and in ways that undermine the ethics of attribution for one's work. We have found ourselves facing challenges that took us away from our organizing efforts in and with communities, and our commitment to generative innovation. When we turned to literature for help in addressing issues, we came up against practices that simply were not fully applicable to our community-focused work and contributed to the difficulties. The literature on open-source methodologies is limited, so we broadened our scope to include issues facing open education, open-source software and open-access publications, which share some similarities with the issues we have encountered with open-source curricula.

### Definition of open source and its connections to human rights

In comparison to intellectual property, open-source licenses use a licensing system that develops a different relationship between the creator, intermediaries and the end users. The open-source movement surfaced from, and along with the growth of the technology sector, as software developers grew frustrated with the limitations of IPR. While developers hoped to collaborate and build off one another's source code for computer programming, the rigid IP legal regime made this difficult and the open-source movement offered a more collaborative alternative (Thiruthy, 2017). According to Thiruthy, “open-source works through a unique licensing system that treats the ‘creation' as property and determines the way in which it can be used and enjoyed. Open-source license creates a relationship between the creator and the user, which is distinct from the way in which contemporary intellectual property right[s] models are used to promote creativity” (2017). Open-source licensing enables more flexible access and use, in comparison to IP model. Proponents of open source view the movement as one motivated by community good, rather than profit (Thiruthy, 2017).

Open educational resources (OER) such as Wikiversity—a platform of learning materials, communities and resulting activities—are examples of open-source, educational resources. Farrow (2016) states that the “moral mission of open education has also found a touchstone in international human rights,” including the Paris Declaration, UDHR, and the IESCR, which all recognize education as a human right, another value espoused by CUSP. Scholars and practitioners have described several benefits of the open educational resources (OER) movement such as: cost savings for users (Henderson and Ostashewski, 2018); increased access to various materials (Mtebe and Raisamo, 2014; Henderson and Ostashewski, 2018); support for more independent learners (Kursun et al., 2014; Henderson and Ostashewski, 2018); collaborative networking and open pedagogy which promotes social scholarship (Hegarty, 2015; Veletsianos and Kimmons, 2012); and achieving social justice goals of poverty reduction and promoting equality (UNESCO, 2019).

Another benefit of the open-source movement has been that learners can take responsibility and become self-motivated for their learning, leading to deeper and more engaged learners, something for which many CUSP members advocate in their own approaches (Sclater, 2010). For example, CUSP has emphasized the importance of personal and collective reflection among managers and all staff of organizations using our materials—especially for trainers and facilitators learning our methodologies—as well as for community members who are participating in the norm change activities (CUSP, 2017). We see self-efficacy as a key component of gender transformative processes, for which the open-source movement appears to advocate.

In addition to Wikiversity's platform for content collaboration, Creative Commons is a set of standardized licenses to support designers and creators to share their work and has become a bedrock of the open-source movement. Creative Commons is an INGO that “empowers people to grow and sustain the thriving commons of shared knowledge and culture” through contextual, inclusive, and sustainable sharing (Creative Commons, 2023). Through their licensing system, Creative Commons provides a free tool for individuals and organizations to grant copyright access for various works. The licenses vary in their conditions, such as requiring attribution, prohibiting the commercial use of works, and forbidding modifications to the content. Some CUSP members have explored the use of Creative Commons, but it has limitations, such as not tracking or collecting licensed material and overall uncertainty around the extent to which licenses are enforced.

Moreover, an aspiration to nurture an open, accessible, and engaging learning environment is not always possible. In a review of the nature and importance of ethics in OER, Farrow (2016) insists that open education falls short of its moral vision, particularly in providing equitable access for all due to differential access to resources, languages, and technology. An OER may be freely available, but someone without access to the internet (or electricity or a computer or phone, a printer, or ink) is not able to benefit from that open access at all. Farrow (2016) distinguishes between normative ethics—what *should* be done—and applied ethics, which is the practical *application* of ethics. So, while open source may appeal to CUSP's normative ethics of humanitarianism and egalitarianism, in practice, we see a different picture in the applied ethics of open source. For instance, although Henderson and Ostashewski (2018) argue that OER may be cost saving, this is not always the case for CUSP, where it also requires a significant amount of our own time to create and then monitor policies for ethical use. Therefore, we cannot conclude that “open” is “good” without analyzing the implications behind “open.”

The advent of Artificial Intelligence (AI) has now taken communications technology one step further, obviating the need for human creators of materials. The companies who created ChatGPT have freely harvested copyright material from thousands of websites, book authors, social media, and other widely available sources, to create its background information base without any notification, acknowledgment or remuneration to the creators of those websites' contents (Sparkes, 2023). This hijacking and rebranding of information, (now also known as “scraping” and sometimes referred to as “copyright at scale” or “plagiarism at scale”) is similar to—and one step further beyond—our experience with the misuse of our methodologies. Moreover, the material being scraped to produce AI materials is predominantly created by white middle class men in the Global North, thereby raising further concerns about the inherent racial, gender, and regional biases in the resulting outputs (Leavy, 2018; Cave and Dihal, 2020; Arora et al., 2023). Discussions around the nature and future of AI have heightened across many sectors, including in the field of global development with a recently adopted UN recommendation on the Ethics of Artificial Intelligence that emphasizes the opportunities and risks of AI, the latter of which describes IP violations, data scraping, and inadequate data protection for individuals (UNESCO, 2022).

The concept of fake news illuminates the contradictions between open source and ethics. “Even if the information is available, open, that does not mean that those who benefit from it can process that data without complying with legal and ethical standards” (Baiasu, 2020). Making material open source has serious implications if not managed responsibly, kept up to date, and attributed to a trusted author, especially when considering the risks of AI adapting or repackaging social norms change materials. In fact, the CEO of OpenAI, which is responsible for creating ChatGPT, supports government regulations on this technology to help mitigate challenges posed by AI, including harassment of women and weaponized disinformation (Bhuiyan, 2023). Just as there are important conversations underway around curbing harmful effects of AI, we believe similar discussions are necessary in the development sector, particularly in light of the many poorly adapted versions of social norm change curricula that are being taken to scale.

### Understanding open source in the context of CUSP's work

Open-source educational resources illustrate the conundrum in which CUSP finds itself: recognizing the exciting possibilities of connecting with learners around the world, but concerned with the disparity created by lack of access to basic information technology hardware, software, and electricity, or to a rich interactive learning environment. This is, in turn, further exacerbated by gendered, racialized, and other structural inequities. For CUSP, open source relates to the decision(s) to share our full methodologies publicly, and CUSP members have differing approaches to the question of open-source access. These differences can be attributed to preferences, organizational structures, and context among CUSP members.

For instance, IRH and Oxfam have open-source materials because of donor requirements and the intent to reach as many people as possible. Specifically, IRH worked in partnership with international and local organizations to develop interventions that could be integrated into ongoing programs and scaled through available resources, based on existing approaches and materials developed and tested by partners. The U.S. government and foundations funding these research initiatives mandated that they be publicly available to all. Developed by consortia led by a Global North university, these implementation toolkits and adaptation guidelines were scalable, focused approaches— somewhat different from the holistic, organic nature of other programs developed earlier by other CUSP members. As part of a university, IRH had not been concerned about their adaptation and replication, viewing it as part of ongoing learning and program development. IRH is not an activist organization, and these approaches did not evolve over years of community-based engagement and learning. Once the research ended, there was no entity concerned with promoting or supporting these approaches. Adaptations have sometimes strayed from the original, yet there have been valuable contributions, including integration of standard approaches to working with fathers to prevent violence against children into INGO country offices, and integration of age-appropriate gender transformative approaches into children's clubs.

On the other hand, Tostan limits access to most of their resources, except for their children's books, a COVID-19 brochure and training materials. Tostan is an international nonprofit headquartered in Thies, Senegal that seeks to empower African communities to bring about sustainable development and positive social transformation based on respect for human rights. Their 3-year Community Empowerment Program (CEP) ignites community dialogue on a wide range of topics through modules focused on democracy, human rights, hygiene, health, and problem-solving. Tostan has not made the curriculum for the CEP available because it is frequently revised based on community feedback and early sharing of the curriculum led to misuse. Because of the pressure early partners felt to implement “short term projects,” early partners chose to extract sessions from the CEP or shortened the facilitator training, resulting in outcomes that were harder to account for than from those of the fully implemented program. Thus, Tostan decided not to share its curriculum in its entirety. Over time, Tostan has made material available to participants from its 10-day sharing seminars, seeing both positive use of the materials—strengthening grant-writing and engaging more meaningfully with community members—as well as some misuse of the materials, including pedagogical modifications that favor lecture over engagement and lack of attribution to Tostan. Ultimately, the risks of keeping the training materials fully open outweighed the benefits.

In comparison, the *Stepping Stones* creators built a principle of shared learning into its dissemination from the outset. Its use in many different contexts over 30 years has seen several adaptations of highly varied quality. *Stepping Stones* was initially developed with diverse donors, with the creators' and publisher's intention of broad use by many global organizations through purchase of the hard copy at a small fee. The profits from sales would then subsidize free hard copies for smaller organizations who could not afford to buy the program or had restricted access to foreign currency. This policy was linked from the outset to the development of an “international community of practice,” where *all* recipients of the material joined this fast-growing database and were actively encouraged to share their experiences around adapting and using the materials in diverse contexts, in a spirit of mutual international sharing and learning. While many partners, large and small, respected this policy through rich collaboration and communication with *Stepping Stones*, over time INGOs and others undermined and disrespected the policy by creating and distributing their own photocopied Portable Document Formats (PDFs). The policy collapsed when one organization adapted the methodology, excluded the original *Stepping Stones* creators from the copyright, and published an adapted PDF version online. As a result, there are now various versions of *Stepping Stones* freely available online, funded by donors whose grantees have not been asked to engage with the creators or follow the adaptation and training guidelines developed by Salamander Trust to help organizations uphold key principles of safe, ethical, or scientifically up to date programming. Resulting programs have been conducted with inadequate training of facilitators and/or inadequate involvement of wider community stakeholders (e.g., Gardsbane and Bukuluki, 2023). Limited or no collaboration or communication with the originators has certainly led to much greater distribution and use of adapted materials, but at what cost to the sexual and reproductive health and rights of their intended participants?

Raising Voices has historically made all material open source in the spirit of collaboration and in service to the field of VAW prevention. Initially, smaller feminist organizations were interested in *SASA!* and used the materials ethically and in direct communication with Raising Voices. However, after the positive results from the *SASA!* RCT emerged, interest ballooned among INGOs, UN agencies and large funders, making it increasingly difficult for Raising Voices to get a clear understanding of the adaptation and use of their materials. There are now multiple versions and adaptations of *SASA!* available online, many of which do not maintain fidelity to the original approach, or the components deemed by Raising Voices to be “essential” to make the approach effective. Yet these materials still use the name and logo of *SASA!*; this causes confusion about the original work, creates safety concerns for communities, breaches good practice in professional acknowledgment of Raising Voices' work and undermines the reputation of *SASA!* when adaptations are low quality. In one case in South Africa—funded by a bilateral donor in a large, multi-country initiative for girls—the three-year community mobilization process was reduced to a two-week training workshop. Several other “adaptations” similarly lacked basic fidelity to the original evidence-based approach, often due to funding issues and pressure for quick programming or scale without sufficient staffing to ensure quality and avoid harm. As a result, *SASA! Together—-*an updated version of *SASA!—-*remains open source but has been gated on the organization's website, with an explicit request that organizations agree to certain ethical conditions of use and share their contact information. This process, however, still relies on an “honor” system and quality control remains difficult given the frequent informal sharing of materials and limited resources available for oversight.

Several CUSP groups have made their materials open source as a matter of principle to serve the field, contributing our experience and learning to the public domain because we see our work not as “ours” but for the collective good. The expectation for many of us was that groups would see, experiment with, and further strengthen the gender transformative movement. However, this has happened to a far lesser extent than hoped, particularly considering current funding and research paradigms prioritize costly RCTs. CUSP has critiqued RCTs extensively, especially regarding their inability to capture complex norms change, high cost, and Western-imposed positivist assumptions (Goldmann et al., 2019; Community for Understanding Scale Up, 2022). Thus, many innovations are not funded because they face a costly measurement barrier to entry, and as such, the “tried and tested” methodologies, including many of CUSP's, continue to dominate.

Further, with open-source materials, CUSP originators have observed how the pressures for quick results with the lowest investment yield the difficulties described above, putting organizations in a bind to find shortcuts such as using an abridged form of our programs, especially when going to scale. When this creates problems in communities or yields poor results, CUSP members are often called in to provide no cost technical assistance to address the concerns. Given these pressures, organizations sometimes produce adapted versions that fundamentally misalign with the principles of the original methodology and their evidence-based impact work, such as reducing time frames, shifting to more “expert” and/or more didactic forms of training, profiting from the materials, excluding reflective sessions that touch the core of uncomfortable truths, reducing time for relationship building, and prioritizing quantity of outputs (e.g., number of people trained) over quality outcomes (e.g., the extent to which social norms have shifted). When implementation styles bear little resemblance to the original methodology, poorly adapted online versions result in potential harm to the people the materials are intended to support. Initiatives may drive violence underground, further encourage men who use violence, raise hopes in the community which can lead to an increase of women reporting violence without the social support needed, and/or undermine community trust for future initiatives.

In addition to the possible harm to participants in these programs, the misappropriation of CUSP methodologies implicates originators. Following misuse of a program, a donor or implementer may conclude that the original methodology is not effective, rather than asking to what extent fidelity was maintained or whether there were other influencing factors. Subsequently, originators often learn only through others that their original work is faulted for poor results; and if they *are* informed, it is to provide emergency technical advisory services to minimize harm. For example, the adaptation of the IMAGE approach in Latin America was met with many challenges. IMAGE's Sisters For Life (SFL) program is a participatory gender and HIV training program fully integrated into routine loan center meetings and delivered alongside microfinance services. The Latin American adaptation had no impact on VAW; the program did not show change in women's attitudes or norms around VAW. This raised questions amongst donors and implementers about the effectiveness of IMAGE methodology. However, the assessment of the adaptation process and implementation revealed misalignment from the original methodology, with SFL's curriculum adapted from 10 one-hour sessions to six 30-min sessions and the facilitators' training was reduced from 2 months to 1 week. Implementation also faced several operational challenges including poor attendance, thus highlighting some of the potential contributing factors to the ineffectiveness of the adapted methodology.

In sum, open source can, in theory, promote equity, aligning with normative ethics of what should be done in circumstances that are built upon mutual respect and authorial integrity. IRH's design for scale at the onset has yielded generative innovation and widespread use of their materials. For other CUSP members, we find that open source can prop up a system of in-and-out programming, burden originators with increased financial and human resource needs, and, worst of all, harm women and communities. Open source does not always yield the type of collaborative field building for which it was intended. Current practices of the open-source movement violate key principles underpinning our vision of feminist scale, including valuing local knowledge and agency, building relationships rooted in collaboration, trust, and care, and fostering an ecosystem that enables interactive and iterative learning.

## Actionable recommendations

The questions surrounding IP and open-source material as it relates to gender transformative social norms change programs impact human lives, particularly for those who already live at the margins of society. When these tools are conceptualized by their originators, there is deliberation, intention-setting, methodological decisions, and rich context informing the design of the approach. There is hope that this creation will have a positive impact in creating happier, healthier, and safer communities. Often, there are safeguards to encourage appropriate use, such as technical support, case studies, MoUs, and/or clear guidance. Misuse, including selective usage of only certain components, and under-resourced adaptation and implementation of the methodology can lead to increased rates of violence, intensified vulnerability, and more.

CUSP's concerns stem from an ecosystem that is not well equipped to support collaboration—particularly a donor system that actively encourages competition and a constant quest for building something “unique,” “innovative,” and “new,” echoing Enlightenment ideology that promotes rigid scientific theory, unimpeded growth, and exploitation.^2^ CUSP members' respective efforts to promote ethical uptake and shared evolution, learning and collaboration around our methodologies are therefore fundamentally undermined and foiled by the inbuilt competitive and individualist ecosystem in which the current global development industry operates.

While there have been attempts by CUSP members to review and advise users of our respective methodologies, there is simultaneously a clear burden placed on originators—who often have fewer staff and capacity—for monitoring fidelity, uptake, and adaptation of the methodologies. One key question remains: *who is responsible for implementing effective and ethical programming?* From our perspective, organizations and the donors who choose CUSP methodologies have a responsibility to implement effective and ethical programming, engaging in necessary due diligence, review of guidance, and communication with originators and successful adaptors.

As such, we pose the following recommendations to stakeholders involved in the gendered norms change ecosystem.

1) Donors:

 a) Practice caution when requiring the specific use of gender-transformative social norms change methodologies in calls for proposals—quality programming requires meaningful design, adaptation and implementation by individuals and organizations who have experience doing the work.
 b) Fund women's rights organizations to embark on their own methodological journeys to create and pilot community and movement-based approaches.
 c) When funding gendered norms change, budget for technical assistance, sufficient training, and ample time for program design, adaptation, and implementation.

2) INGOs and IDCs:

 a) Honor the existing IP of gendered norms change curricula and programs that are adapted.
 b) Uphold safety and monitor for backlash throughout implementation.
 c) Be accountable to the broader movement, including through transparent and equitable funding for implementing organizations, planning, and funding technical assistance (ideally from originators or individuals/groups they recommend), and documentation and dissemination of process as well as results.
 d) In contracts for implementation or technical assistance, ensure IP for any innovation remains with the implementing organization and/or originator.
 e) Maintain realistic expectations about programmatic impact if implementation is not fully funded, cut short, and/or is poorly adapted.

3) Researchers

 a) Embrace a social-justice driven IP as opposed to a market-driven one.
 b) Honor the existing IP of gender norms change curricula and programs that are adapted.
 c) Address and support the rich and complex challenges and opportunities of women's lives beyond the inevitably narrow results of RCTs through opportunities such as practice-based learning and implementation science.
 d) Acknowledge the value of implementing organizations' experience and contribution to research and knowledge building by committing to equitable partnership, transparency, and visibility and voice of community members and organizational partners (including through co-authorship).
 e) Commit to popularizing results of all studies to promote usability by activist groups, including those innovating and adapting social norm change programming.

## Discussion

Many of our methodologies entail a process of deep learning and/or unlearning for all involved: community members, organizations, trainers, facilitators, and ourselves. Just as we encourage others to reconsider the attitudes, beliefs and practices that have been so deeply held, we invite funders, researchers, multilateral and bilateral agencies, INGOs, NGOs and IDCs to challenge their own social norms around scaling: rethink the ways the broader field enables the misuse of methodologies through the prioritization of quantity (of people reached) over quality (of programming) and of competition over collaboration.

At the same time, we are engaging in self-reflection as we come up against contradictions such as our methodologies being derived from other resources—the result of intentional research and concepts that preceded the program–and the contributions of community members to these programs. CUSP methodologies have their own histories that are built upon various theoretical and practice-based resources, such as Paulo Freire's approach to conscientization, Augusto Boal's Theater of the Oppressed, and the distinct types of power expanded upon in *SASA! Together* (e.g., Townsend, 1999). Moreover, this work involves the iterative engagement of those not formally within our organizations–community members who are sometimes, although not always, re-engaged to see how these methodologies adapt, spread, and evolve. If CUSP views scale through the lens of feminist movement building, we must contemplate how too much oversight can stifle this spirit. One CUSP member described conversations on IPR and open source as a river that continues to move, evolve, and flow, collecting (and even removing) material naturally as it travels.

In our collective discussions over the past year to develop this paper, we have also seen how issues of IP and open source are growing ever more topical and pressing with the rapid rise of AI and new forms of technology-facilitated VAW. Thinking through these terms and their associated practices is part of our work as innovators and practitioners in the global development sector who aspire to “do good.” Eventually, we find ourselves returning to our fundamental feminist values. First, we must continually examine power in all its diverse and intersecting forms. Second, we believe that sharing our collective experiences can inform both future theory and praxis. Next, we believe that feminist partnerships—through mutual collaboration and shared purpose—can help advance this complex work to embrace innovation while minimizing harm. And lastly, that we must always maintain our deep, political commitment to creating safer realities for women and girls arounds the world.

## Footnotes

^1^CUSP's definition of methodologies not only refers to the finalized products, training guides and curricula material resulting from programs, but also embodies the feminist ethics around shared power, accountability, and commitment to safety that provides the requisite foundation for these approaches to truly flourish in their intended manner.

^2^CUSP has previously discussed the consequences of a western philosophical legacy in global development, which “justified the thirst for expansion, control, extraction and new knowledge”; and supported colonial expansion of economic and political power under assumptions of superiority from Western countries. For more, read *https://raisingvoices.org/wp-content/uploads/2022/01/CUSP_FeministScalePaper-2.pdf*
